# The diagnostic value of metagenomic next⁃generation sequencing in infectious diseases

**DOI:** 10.1186/s12879-020-05746-5

**Published:** 2021-01-13

**Authors:** Hongxia Duan, Xuan Li, Aihong Mei, Ping Li, Yang Liu, Xiaofeng Li, Weiwei Li, Changhui Wang, Shuanshuan Xie

**Affiliations:** 1 Department of Respiratory Medicine, Shanghai 10th People’s Hospital, Tongji University School of Medicine, #301, Mid Yanchang Rd, Shanghai, 200072 China; 2 Department of Emergency, Shanghai 10th People’s Hospital, Tongji University School of Medicine, Shanghai, 200072 China; 3 Department of Geriatrics, Shanghai 10th People’s Hospital, Tongji University School of Medicine, Shanghai, 200072 China

**Keywords:** Next-generation sequencing, Sensitivity, Diagnostic, Infection, Survival

## Abstract

**Background:**

Although traditional diagnostic techniques of infection are mature and price favorable at present, most of them are time-consuming and with a low positivity. Metagenomic next⁃generation sequencing (mNGS) was studied widely because of identification and typing of all pathogens not rely on culture and retrieving all DNA without bias. Based on this background, we aim to detect the difference between mNGS and traditional culture method, and to explore the relationship between mNGS results and the severity, prognosis of infectious patients.

**Methods:**

109 adult patients were enrolled in our study in Shanghai Tenth People’s Hospital from October 2018 to December 2019. The diagnostic results, negative predictive values, positive predictive values, false positive rate, false negative rate, pathogen and sample types were analyzed by using both traditional culture and mNGS methods. Then, the samples and clinical information of 93 patients in the infected group (ID) were collected. According to whether mNGS detected pathogens, the patients in ID group were divided into the positive group of 67 cases and the negative group of 26 cases. Peripheral blood leukocytes, C-reactive protein (CRP), procalcitonin (PCT) and neutrophil counts were measured, and the concentrations of IL-2, IL-4, IL-6, TNF-α, IL-17A, IL-10 and INF-γ in the serum were determined by ELISA. The correlation between the positive detection of pathogens by mNGS and the severity of illness, hospitalization days, and mortality were analyzed.

**Results:**

109 samples were assigned into infected group (ID, 92/109, 84.4%), non-infected group (NID, 16/109, 14.7%), and unknown group (1/109, 0.9%). Blood was the most abundant type of samples with 37 cases, followed by bronchoalveolar lavage fluid in 36 cases, tissue, sputum, pleural effusion, cerebrospinal fluid (CSF), pus, bone marrow and nasal swab. In the ID group, the majority of patients were diagnosed with lower respiratory system infections (73/109, 67%), followed by bloodstream infections, pleural effusion and central nervous system infections. The sensitivity of mNGS was significantly higher than that of culture method (67.4% vs 23.6%; *P* < 0.001), especially in sample types of bronchoalveolar lavage fluid (*P* = 0.002), blood (*P <* 0.001) and sputum (*P* = 0.037), while the specificity of mNGS was not significantly different from culture method (68.8% vs 81.3%; *P* = 0.41). The number of hospitals stays and 28-day-motality in the positive mNGS group were significantly higher than those in the negative group, and the difference was statistically significant (*P* < 0.05). Age was significant in multivariate logistic analyses of positive results of mNGS.

**Conclusions:**

The study found that mNGS had a higher sensitivity than the traditional method, especially in blood, bronchoalveolar lavage fluid and sputum samples. And positive mNGS group had a higher hospital stay, 28-day-mortality, which means the positive of pathogen nucleic acid sequences detection may be a potential high-risk factor for poor prognosis of adult patients and has significant clinical value. MNGS should be used more in early pathogen diagnosis in the future.

## Background

Infectious diseases are a leading cause of morbidity and mortality worldwide and spread quickly. As the first-line of pathogen detection, microbiology laboratory plays an important role in infection control by means of microscopic examination, culture, identification, drug sensitivity and so on [1]. However, the limitation of molecular diagnosis and genotyping methods remain that pathogens are undetected in up to 60% of cases [2–4]. Failure to identify pathogens in time may delay the precise treatment of antibiotics, leading to unnecessary use of broad-spectrum antibiotics, inducing resistance, and increasing medical costs [5].

With the completion of the human genome project in the early twenty-first century and the rapid development of sequencing technology, high-throughput and low-cost second-generation sequencing technology emerged [6]. It had been used in whole genome sequencing, whole exome sequencing, macro gene sequencing and so on, among which metagenomic next⁃generation sequencing (mNGS) was studied most widely. The advantage of mNGS lies in the single run to obtain the sequence information of microbial nucleic acid fragments, through analysis and comparison of which to detect all microbial species and sequence [7]. Besides, mNGS can be used for the identification and typing of all pathogens because mNGS does not rely on culture and retrieve all DNA without bias [8]. Based on mNGS results, antimicrobial resistance, virulence, typing and other information can be used for epidemic investigation. It lays a theoretical foundation for the investigation of infectious diseases outbreak in hospital. Therefore, this technology may play a huge role in infection prevention and medical microbiology laboratory.

Thus, based on microbiome sequencing technology, we compared the sensitivity and specificity of mNGS method and traditional culture method to detect pathogens, and discussed the influence of mNGS detection results on the severity and prognosis of patients with infection in our study.

## Methods

### Study patients

**Fig. 1:**
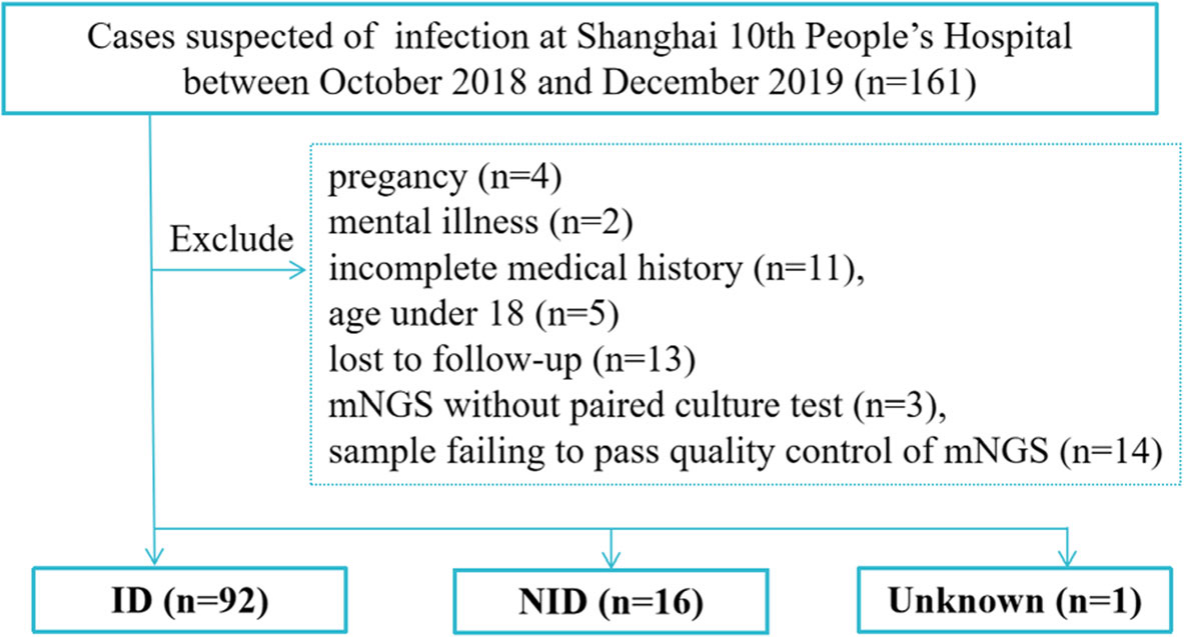
Flow diagram of cases inclusion and exclusion

We retrospectively reviewed 161 cases suspected of acute or chronic infection from respiratory and critical care medicine department, geriatric department, emergency intensive care unit and emergency department at Shanghai 10th People’s Hospital in Shanghai, China, between October 2018 and December 2019. Excluding patients with pregnancy, mental illness and under the age of 18, 109 samples were included in our study and for analysis and then they were categorized into 3 groups, infectious disease (ID) group, noninfectious disease (NID) group, and unknown group according to final diagnosis. Specimens were subjected to mNGS testing (BGI, Intertek, Biotecan, China) and regular clinical microbiological assay in a pairwise manner and final diagnosis was determined by clinicians based on both of them and imaging, clinical feature of patients. Meanwhile, clinical data of all enrolled patients, including complete blood count, C-reactive protein (CRP), procalcitonin (PCT), neutrophil count, interleukin (IL)-2, IL-4, IL-6, Tumor Necrosis Factor-α (TNF-α), IL-17A, IL-10 and Interferon-γ (INF-γ) were collected. The flow diagram of cases inclusion and exclusion was shown in Fig. 1. This research had been approved by the ethics committee of the 10th People’s Hospital affiliated to Tongji University (No. SHSY-IEC-4.1/20–21/01).

### Metagenomic next-generation sequencing and analysis

**Fig. 2:**
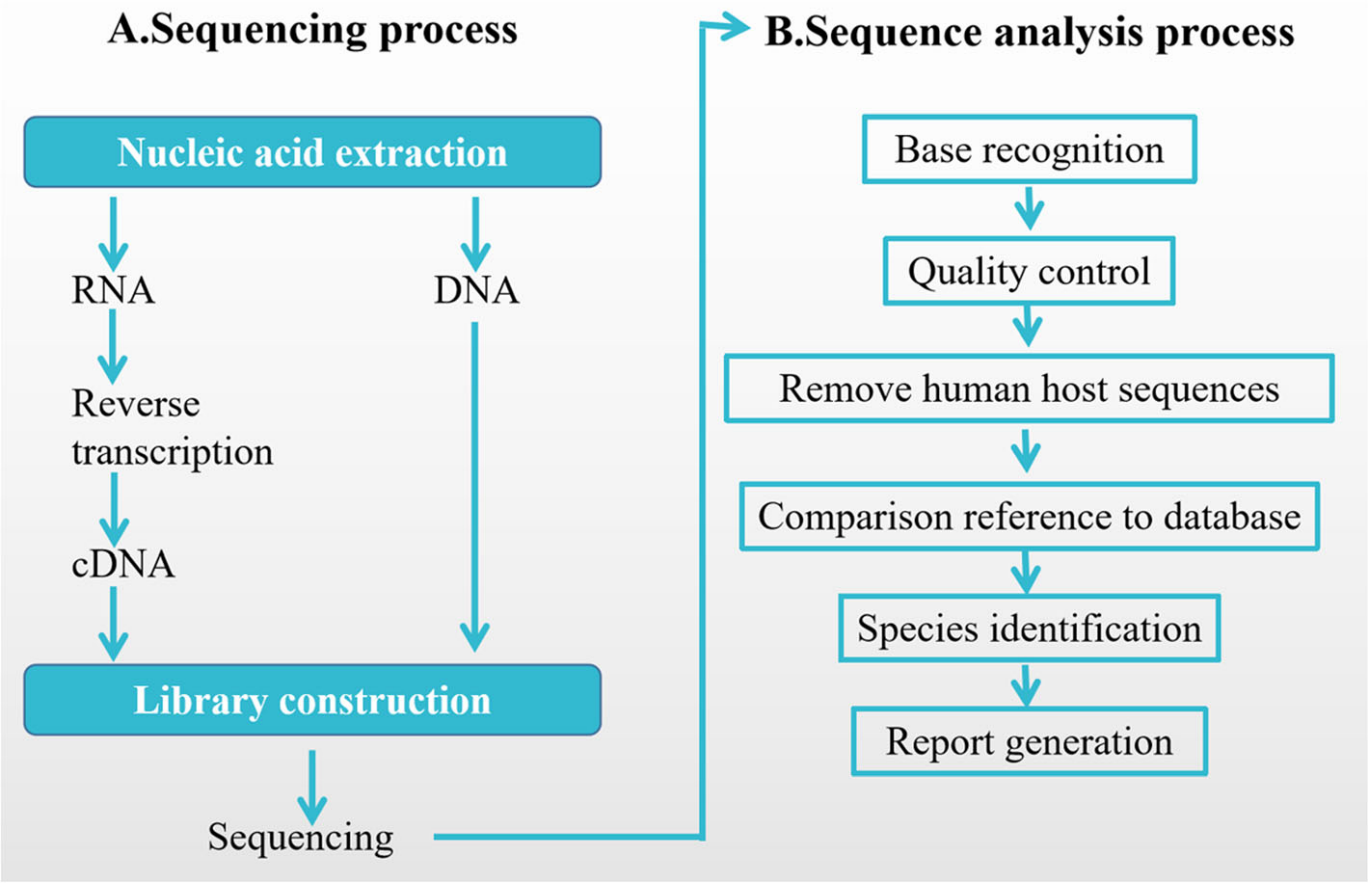
Flow diagram of Metagenomic Next-generation Sequencing and Analysis

Nucleic acid detection and sequencing were performed based on BGISEQ-50 platform (BGI-Tianjin, Tianjin, China) in this research. After the sample was taken, nucleic acid was extracted, the library was built and sequenced, and finally the data was analyzed by using the microbiome database (ftp://ftp.ncbi.nlm.nih.gov/genomes/). The experimental process was shown in Fig. 2.

#### Sample processing and library construction (Fig. 2a)

For infected patients or patients with fever of unknown cause, infected site samples or blood were collected according to standard procedures. Each blood, bronchoalveolar lavage Fluid (BALF) or urine sample was at least 5 ml (ml) and at least 3 ml of each sample of cerebrospinal fluid, sputum, or other sterile liquid. Blood must be collected in anticoagulant tube and stored at room temperature, the protective agent in anticoagulant tube is Ethylene Diamine Tetraacetic Acid (EDTA) anticoagulant and special deoxyribonucleic acid (DNA) protective agent. Other samples were collected in sterile tube and stored at − 80 °C. Blood was transported at room temperature, and other sterile samples were transported in drikold. Since most of the collected samples contain pathogenic pathogens, they were inactivated (56 °C, 30 min) before nucleic acid extraction. In addition to this, blood samples were centrifuged to separate plasma and leukocytes when intracellular bacterial infection was particularly suspicious by clinicians. Sputum samples were liquefied by using 0.1% dithiothreitol (DTT) for 30 min at room temperature after inactivation [5]. After that, DNA were extracted by TIANamp Micro DNA Kit (DP316, Tiangen Biotech) according to the manufacturer’s recommendation. DNA libraries were constructed in steps of DNA fragmentation by enzyme digestion, DNA supplementation terminal, dA tail and sequencing common connector connection. The constructed DNA library was used to obtain the sequence data of DNA fragments by gene sequencing instrument, and the results were analyzed by biological information software. Each trial included internal, negative and positive controls. Internal parameters is a specific molecular tag that is placed in the sample before nucleic acid extraction to track the entire process and to control the quality of DNA. The detection results of negative control products should be no pathogens detected. If there are relevant pathogens detected, it indicates that there may be DNA pollution sources in the environment. Positive contained specific microbic DNA.

#### Bioinformatic analysis (Fig. 2b)

##### Quality control

A. Sequencing subtracted of human host sequences need to be above 90%; B. Reads of microbial detection sequences need to be longer than 50 bp and the effective sequencing data volume should not be less than 20 M without removing the human genome component.

##### Data filtering

In order to obtain high quality sequence data, the qualified data was further filtered by bioinformatics analysis to remove low quality sequences. FASTQ format was used for analysis. The initial pretreatment steps include low quality read filtering, low- complexity read filtering and adapter trimming. Host subtraction was performed by mapping to host genome and/or transcriptome. The remaining unmapped reads are aligned directly with large reference databases, such as the National Center for Biotechnology Information (NCBI) GenBank database.

##### Sequences alignment

The filtered sequences were compared with the reference sequences in the pathogen database, which covers bacteria, fungi, viruses, protozoa and other pathogenic microorganisms. According to the final results of pathogen comparison, all parameters of detected pathogens were calculated, including sequence number, relative abundance, genome coverage and depth, etc.

##### Report generation

The species listed in the report were all the microorganisms detected in this test. They were classified by bacteria, viruses, fungi, parasites, mycoplasma, chlamydia and rickettsia. They were ranked from high to low according to their reads and the relative content of the former is higher. When the report goes to the clinic, whether the suspected pathogen detected is related to infection from the clinical dimension was judged, and the final diagnosis was determine by combining the detection parameters.

### Determination of cytokines

Detection of TNF-a, IL-2, IL-4, IL-6, IL-8, IL-10, IL-17A and INF-r in serum was by solid phase, sandwich and chemiluminescence using the IMMULITE/IMMULIE 1000 analyzer. The analyzer and chemiluminescence kit were both from SIEMENS, Germany. The processed specimens were sent to the analyzer for testing according to the manufacturer’s instructions, and the corresponding cytokine concentrations were recorded.

### Cell classification and count detection

Cells were classified using the automatic flow cytometer (Thermo Fisher SCIENTIFIC, American) and divided into total white blood cells, neutrophil count, CD4+ T cell count, CD8+ T cell count, B cells, and NK, T cell count.

### Statistical analysis

Comparative analysis was conducted by Pearson χ2 test and t test. Data analysis was performed by using SPSS 22.0 software. *P* values < 0.05 were considered significant, and all tests were 2-tailed. Logistic regression analysis explored the risk factors associated with positive detection of mNGS.

## Results

### Sample and patient characteristics

**Fig. 3:**
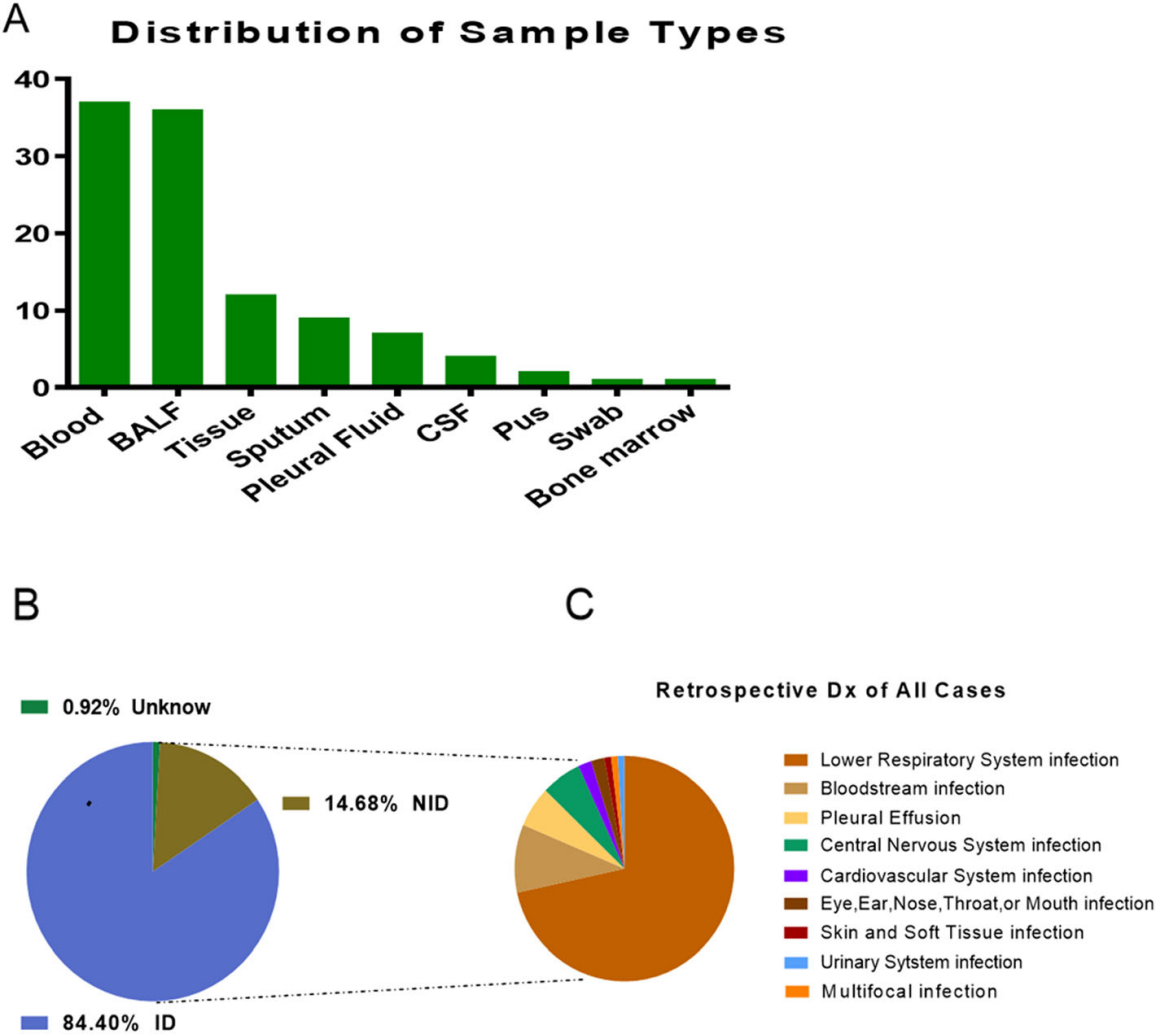
Patients composition and samples types. **a**. In samples of this study, 33.9% were from blood which was the most, 33.0% from BALF, 11.0% from tissue and the others were from sputum (8.3%), pleural fluid (6.4%), CSF (3.7%), pus (1.8%), bone marrow (0.9%) and nasal swab (0.9%). **b**. Patients were subdivided into ID (92/109, 84.4%), NID (16/109, 14.7%) and unknown (1/109, 0.9%) groups according to their diagnosis by conventional technique. **c**. Infection sites of patients in ID group. Most were respiratory system infections (73/109, 67.0%) and followed by bloodstream infections (10/109, 9.17%), pleural effusion (6/109, 5.50%), central nervous system infections (6/109, 5.50%), cardiovascular system infection (2/109,1.83%), eye, ear, nose, throat, or mouth infection (2/109,1.83%), skin and soft tissue infection (1/109, 0.92%), multifocal infection (1/109, 0.92%), urinary system infection (1/109, 0.92%). Abbreviations: CSF, cerebrospinal fluid; BALF, bronchoalveolar lavage fluid

Demographic features of the patients were provided in Table 1. 87 males and 22 females participated in our study, whose average age was 61 years old, average length of stay was 17.5 days and the case fatality rate were 11.9%. Most (37/109, 33.9%) of our samples were from blood, 36 of 109 (33.0%) were from BALF, 12 of 109 (11.0%) were from tissue and 9 (8.3%) of 109 were from sputum, followed by pleural fluid (7, 6.4%), CSF (4, 3.7%), pus (2, 1.8%), bone marrow (1, 0.9%) and nasal swab (1, 0.9%) (Fig. 3a). In the study cohort, 92 (84.4%) patients diagnosed with confirmed pathogens by clinicians were assigned to ID group. The remaining specimens were subdivided into the NID (16/109, 14.7%) and unknown (1/109, 0.9%) groups (Fig. 3b). There were no statistical differences between ID group and NID group in age, gender, length of stay and case fatality rate (*p* > 0.05 in all). Most patients were diagnosed with respiratory system infections (73/109, 67.0%), followed by bloodstream infections (10/109, 9.17%), pleural effusion (6/109, 5.50%) and central nervous system infections (6/109, 5.50%) as shown in Fig. 3c.

**Table 1:** Demographic characteristic of samples

	Total	ID	NID	Unknown	*P* value between ID & NID
Samples amount, n (%)	109 (100%)	92 (84.40)	16 (14.68)	1 (0.92)	/
Age, average years (range)	61.02 (25–95)	60.26 (25–95)	66 (40–90)	61(/)	0.43
Gender,male,n (%)	87 (79.82)	74 (80.43)	12 (75.00)	1 (100%)	0.62
Length of stay, average days (range)	17.53 (1–70)	16.88 (1–70)	20.87 (6–61)	22(/)	0.31
case fatality rate, %	11.93	13.04	6.25	0	0.39

**Abbreviations:** *ID* infectious disease, *NID* noninfectious disease

### Diagnostic performance comparison of mNGS and culture

#### Comparison of diagnostic performance for differentiating ID from NID

**Fig. 4:**
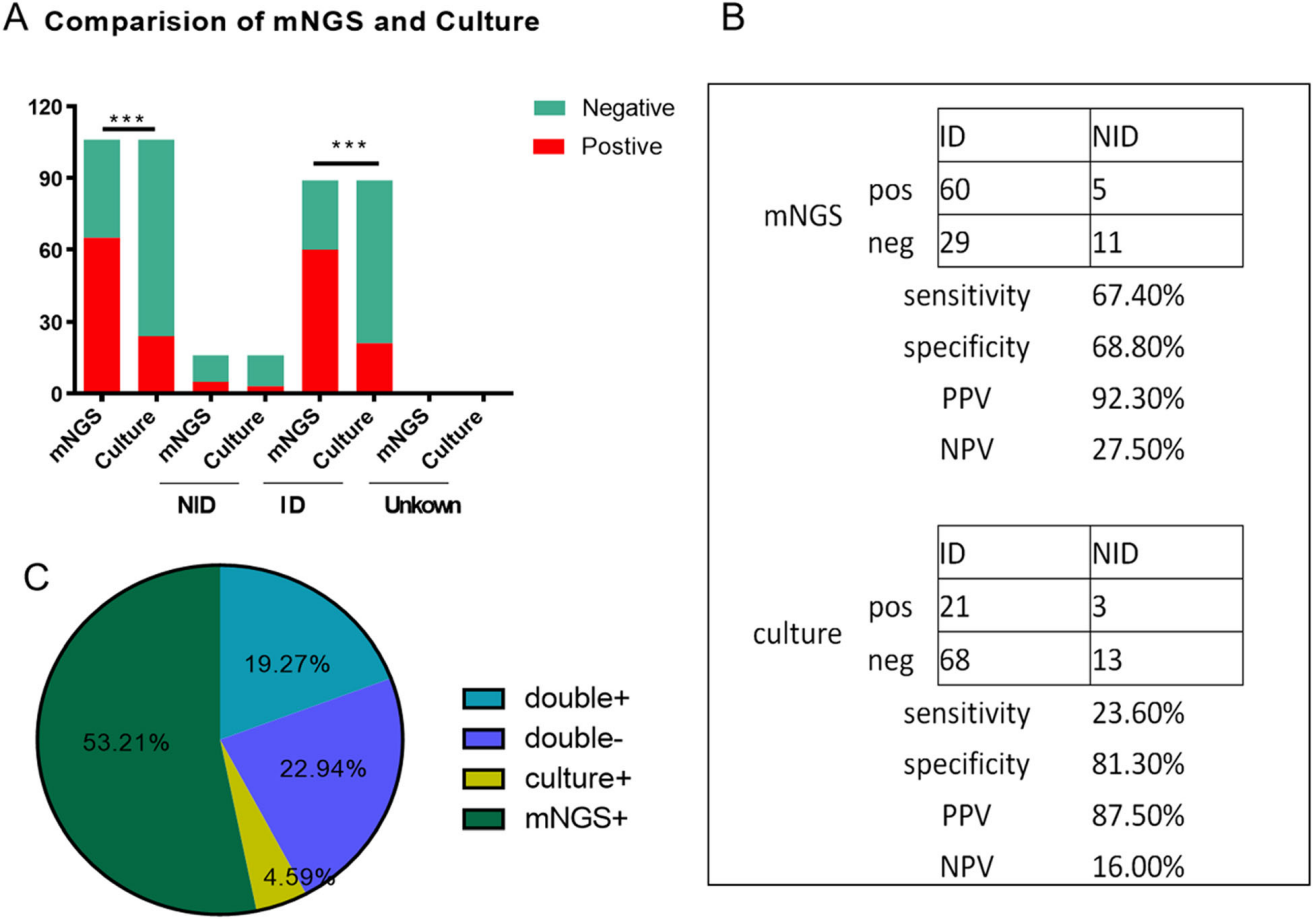
Diagnostic Performance Comparison of mNGS and Culture. **a**. Positive and negative cases in all, ID, NID and unknown group of mNGS and the culture, respectively. There were statistical differences between mNGS and culture of all (*P* < 0.01) and of ID group (*P <* 0.01), but no differences in NID and unknown group for the limited amounts(*P* > 0.05). **b**. Contingency tables showed the sensitivity and specificity of mNGS were 67.4 and 68.8%, while those of culture were 23.6 and 81.3%. mNGS increased the sensitivity in comparison with that of culture (*P <* 0.001) while there were no differences in specificity between them (*P* = 0.41). **c**. Pie chart demonstrated the positivity distribution of mNGS and culture for all samples from 3 groups. 53.21% were positive by mNGS, 4.59% by culture, 19.27% by both and 22.94% were both negative. Abbreviations: NPV, negative predictive values; PPV, positive predictive values

The cases of mNGS and culture tests in this study were illustrated in Fig. 4a. In the chi-square test of positive rate, there were statistical differences between mNGS and culture of all and of ID group, but no differences in NID and unknown group for the limited amounts. 105 samples were included for further study to compare the diagnostic efficiency for differentiating ID from NID. The positive predictive values and negative predictive values of diagnosing infectious disease by mNGS were 92.3 and 27.5%, respectively. The positive likelihood ratio and negative likelihood ratio being 2.16 and 0.47. The results showed that mNGS increased the sensitivity rate (positive number in ID/ID number) by approximately 44% compared with that of culture (67.4% vs 23.6%; *P* < 0.001) and decreased the specificity rate (negative number in NID /NID number) by 12.5% compared with that of culture (68.8% vs 81.3%; *P* = 0.41) (Fig. 4b).

#### Concordance between mNGS and culture for pathogen detection

In this study, mNGS and culture were both positive in 21 of 109 (19.3%) cases and were both negative in 25 of 109 (22.9%) cases. There were 58 cases (53.2%) were positive by mNGS only and 5 (4.6%) were positive only by culture. The 2 results in double-positive cases were completely matched (overlapped of all pathogens) in 3 of 21 and totally mismatched (overlapped of no pathogen) in 3 of 21 (Fig. 4c). The remaining 15 cases were found to at least one but not all overlapped of pathogens in polymicrobial results, which defined as “partly matched”.

#### “False positives” and “false negatives” of mNGS

In the ID group, three culturable pathogens were missed by mNGS. Among the three “mNGS false-negative” samples, there were 2 culture results paradoxical with clinical diagnosis, the other 1 was completely unidentified by mNGS. At the same time, the possible reasons for the 7 cases of “mNGS false-positive” in the NID group included potential concomitant infection with NIDs (3/7), overinterpretation (3/7) and unknown (1/7) (Table 2).

**Table 2:** “False Positives” and “False Negatives” of mNGS

Pathogens Detected Only by mNGS in the NID Group				
Sample No.	Specimen source	Diagnosis	mNGS result	Possible explanation
2	BALF	Hematencephalon	Acinetobacter baumannii, Klebsiella, Enterococcu	Unknown
33	Blood	Lymphoma	Pseudomonas, CMV	Potential cause of lymphoma
62	Blood	Aplastic anemia	Acinetobacter baumannii, Enterococcu	Overinterpretation
67	Blood	myelofibrosis	Phycomyces blakesleeanus	Overinterpretation
74	Pleural Fluid	Pleural effusion	Fusobacterium nucleatum, Streptococcus constellatus, Porphyromonas gingivalis	Potential cause of inflammation
86	Blood	Ulcerative Colitis	Porphyromonas gingivalis, HSV	Potential cause of inflammation
88	Blood	Lung cancer	*Saccharomyces cerevisiae*	Overinterpretation
**Culturable Pathogens Missed by mNGS in the ID Group**				
**Microbe**		**Count**	**Possible explanation**	
MTB		2	Positive Not Detected	
Pseudomonas		1	Microbes “Weak”	

**Abbreviations:** *mNGS* metagenomic next-generation sequencing, *ID* infectious disease, *NID* noninfectious disease, *CMV* metagenomic next-generation sequencing, *HSV* herpes simplex virus, *MTB Mycobacterium tuberculosis*

### Comparison of mNGS and culture testing by pathogens and samples

#### Comparison analysis at the pathogen-type level

**Fig. 5:**
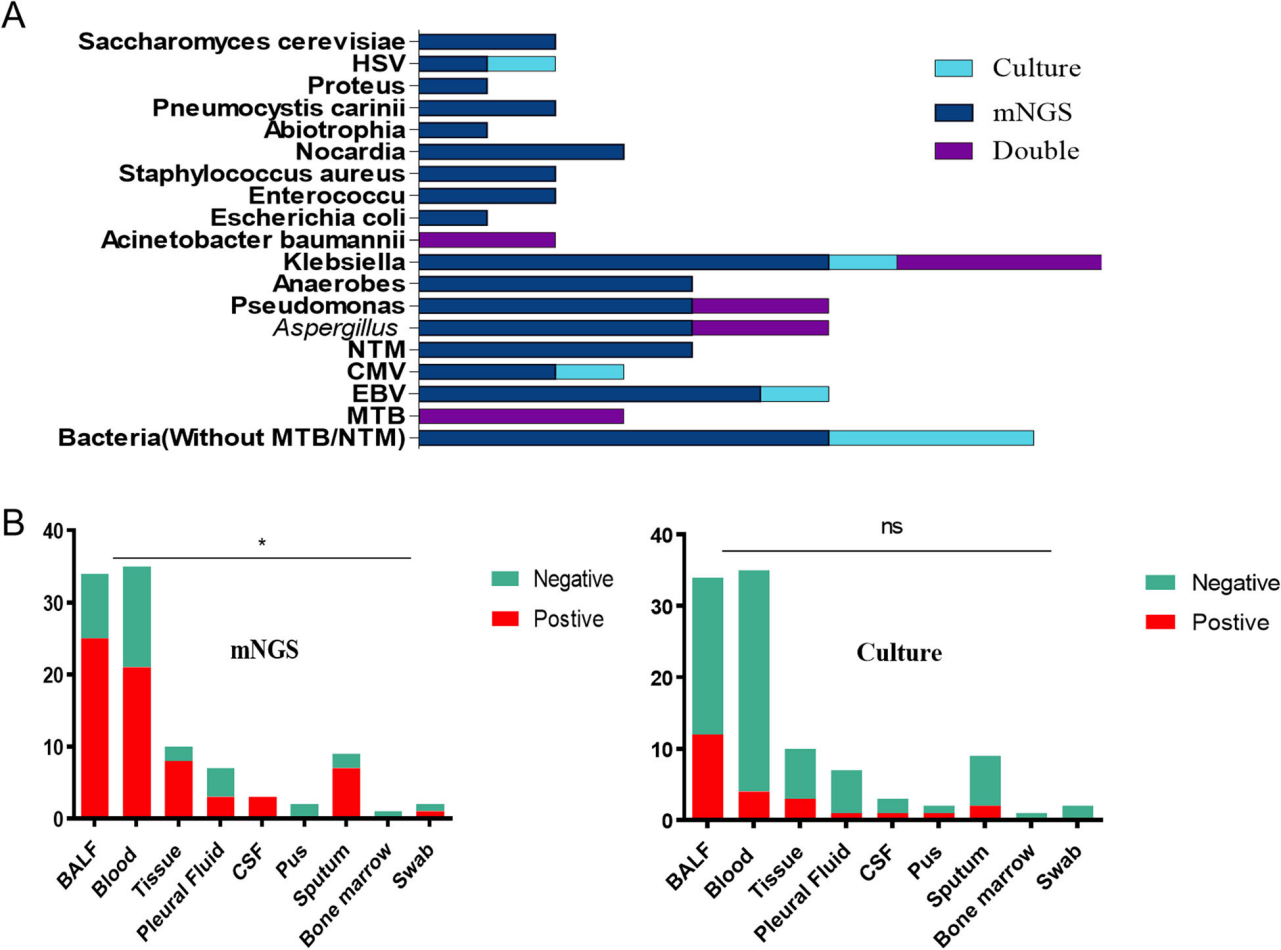
The overlap of positivity between mNGS and culture in pathogen and sample types. **a**. 19 pathogens detected in ID group with their corresponding frequencies were showed in histograms. Klebsiella, bacteria without MTB/NTM, EBV, CMV, NTM, Anaerobes, Saccharomyces cerevisiae, Proteus, Pneumocystis carinii, Abiotrophia, Nocardia, *Staphylococcus aureus*, Enterococcu and *Escherichia coli* demonstrated a trend of higher positivity rate in mNGS than that in culture with no statistical differences (*P* > 0.05). Acinetobacter baumannii and MTB were found equally in two groups. **b**. The overall sensitivity of mNGS in the different sample types were significantly different (*P* = 0.03) while sample types did not affect the sensitivity of pathogens in culture. Interestingly, especially in the types of BALF, blood and sputum samples, mNGS had significantly higher sensitivity than the culture (*P* = 0.002 for BALF, *P <* 0.001 for blood, *P* = 0.037 for sputum). Abbreviations: BALF, bronchoalveolar lavage fluid; CSF, cerebrospinal fluid; mNGS, metagenomic next-generation sequencing; HSV, herpes simplex virus; CMV, cytomegalovirus; EBV, Epstein-Barr virus; MTB, *Mycobacterium tuberculosis*; NTM, nontuberculous mycobacteria; ns, no significant difference

Klebsiella (10/69) was the most commonly detected pathogen among the 69 microbes isolated in mNGS and culture testing, followed by bacteria without MTB/NTM (9/69), Aspergillus (6/69), Pseudomonas (6/69) and EBV (6/69) (Fig. 5a). The percentage of mNGS-positive samples observed to have a higher yield rate than that of culture, but the differences were not significant (*P* > 0.05) in terms of Klebsiella, bacteria without MTB/NTM, EBV, CMV due to the small sample size. In Acinetobacter baumannii (*n* = 2) and MTB (*n* = 3), the number of mNGS-positive samples was equally with that of culture-positive samples. While only mNGS indicated positive results in NTM (*n* = 4), Anaerobes (*n =* 4), *Saccharomyces cerevisiae* (*n =* 2), Proteus (*n =* 1), Pneumocystis carinii (*n =* 2), Abiotrophia (*n =* 1), Nocardia (*n =* 3), *Staphylococcus aureus* (*n =* 2), Enterococcu (*n =* 2) and *Escherichia coli* (*n =* 1).

#### Comparison analysis at the sample-type level

In the types of BALF, tissue, blood and sputum samples, mNGS detection had significantly higher sensitivity than the culture method (*P* = 0.002 for BALF, *P* = 0.025 for tissue, *P* < 0.001 for blood, *P* = 0.018 for sputum), and the overall sensitivity of mNGS in the sample types was significantly different (*P* = 0.03). In the types of pleural fluid, CSF, pus, bone marrow and nasal swab, there were no significant differences in sensitivity between two methods (*P* > 0.05). In addition, in the culture method, the positive rate in BALF was higher than that in the whole blood (*P* = 0.019), and there was no difference in the overall sensitivity of the culture method in the sample type, as shown in Fig. 5b.

### Comparison of infection indexes in positive and negative group by mNGS in ID

#### Classification and counting of leukocyte and lymphocyte in positive and negative group by mNGS

In this study, complete blood count, CRP and PCT tests were examined on the day of examination of pathogenic microorganisms to determine the differences in the total number of white blood cells, lymphocytes and neutrophils between the positive group and the negative group by mNGS. The results showed (Table 3) that there were no statistically differences in leukocyte and lymphocyte between positive and negative groups by mNGS (*P* > 0.05).

**Table 3:** The counts of WBC, Cytokines and lymphocytes in positive and negative groups by mNGS

	Positive	Negative	P
**Cytokines pg/ml**			
IL-2	100.35 + 68.21	1.31 + 0.94	0.511
IL-4	2.74 + 0.41	1.52 + 0.94	0.206
IL-6	70.8 + 18.27	68.96 + 33.18	0.964
TNF-α	2.48 + 0.42	2.26 + 1.32	0.842
IL-17a	13.77 + 2.35	10.45 + 8.01	0.592
IL-8	1154 + 0	–	–
IL-10	26.14 + 7.75	8.29 + 3.33	0.044
IFN-γ	8.91 + 1.89	13.59 + 6.92	0.361
**Cellular Immunity %**			
CD4/CD8	1.42 + 0.23	2.06 + 0.44	0.185
Th cell	35 + 3.36	43.83 + 5.75	0.201
Ts cell	68.06 + 3.07	66.67 + 3.64	0.18
NK cell	15.71 + 2.17	15.5 + 1.89	0.958
B cell	13.53 + 2.94	12.83 + 3.73	0.899
T cell	68.06 + 3.07	66.67 + 3.64	0.958
WBC ^×^109	8.32 + 0.52	7.36 + 0.48	0.283
Neu^×^ 109	6.99 + 0.58	5.38 + 0.48	0.109
PCT ng/ml	0.34 + 0.17	3.42 + 3.32	0.112
CRP mg/l	87.63 + 8.32	63.61 + 13.47	0.129

**Abbreviations:** *mNGS* metagenomic next-generation sequencing, *WBC* white blood cells, *IL-* interleukin-, *IFN-γ* Interferon-γ, *TNF-α* Tumor Necrosis Factor-α, *CD4* Cluster of Differentiation 4 receptors, *CD8* Cluster of Differentiation 8 receptors, *Th* helper T cell, *Ts* suppressor T cell, *NK* natural killer cell, *Neu* neutrophil, *PCT* procalcitonin, *CRP* C-reactive protein

#### Comparison of cytokine concentrations in positive and negative group by mNGS

In order to explore the correlation between the status of immune function in patients and the positive results of pathogen examination, this study detected and analyzed the peripheral blood (TNF-a, IL-2, IL-4, IL-6, IL-8, IL-10, IL-17A and INF-r) in infected patients. The results indicated that the peripheral blood concentrations of IL-10 in the positive group was higher than that in the negative group, and the differences were statistically significant (*P* = 0.044), while other cytokine showed no difference between groups as shown in Table 3.

### Analysis of correlative factors for positive result of pathogen extraction by mNGS

In order to further explore the related risk factors of positive mNGS test in infected patients, this study used Logistic multivariate regression analysis to analyze the patients’ information and whether the pathogen was detected in the patients. After the confounding factors were removed, the variables that were significant for detection was age (*P* = 0.037, OR:1.076, 95% CI:1.005–1.152), which promoted the detection of pathogens (Table 4).

**Table 4:** The analysis of the relevant factors of pathogens DNA positive in patients

Values	B	SE (B)	Wald X^2^	P	OR	95% CI
Age	0.073	0.035	4.367	0.037	1.076	1.005–1.152
Sex	−0.545	1.157	0.222	0.637	0.58	0.06–5.601
Read Number	−2.371	0.599	15.677	0.000	0.093	0.029–0.302
HOD	−0.028	0.061	0.216	0.642	0.972	0.863–1.095
Survival Time	−0.007	0.005	1.888	0.169	0.993	0.983–1.003
**Cytokines pg/ml**						
IL-2	0.171	1.115	0.023	0.878	1.186	0.133–10.553
IL-4	−1.299	0.893	2.116	0.146	0.273	0.047–1.57
IL-6	−0.005	0.019	0.077	0.781	0.995	0.957–1.033
TNF-α	−0.374	0.373	1.003	0.316	0.688	0.331–1.430
IL-17a	0.202	0.137	2.165	0.141	1.223	0.935–1.6
IL-10	−2.64	0.206	1.639	0.201	0.768	0.513–1.151
IFN-γ	0.09	0.071	1.606	0.205	1.095	0.952–1.259
**Cellular Immunity %**						
CD4/CD8	−0.488	0.965	0.256	0.613	0.614	0.093–4.067
Th cell	0.318	0.296	1.151	0.283	1.374	0.769–2.454
Ts cell	0.244	0.317	0.589	0.443	1.276	0.685–2.377
NK cell	−0.223	0.211	1.121	0.29	0.800	0.529–1.209
B cell	−0.26-	0.245	1.172	0.279	0.767	0.227–1.475
T cell	0.5485	0.478	1.315	0.252	0.578	0.475–1.239
WBC ^×^109	−0.123	1.228	0.01	0.92	0.884	0.08–9.819
Neu^×^109	0.141	1.39	0.01	0.919	1.151	0.076–17.535
PCT ng/ml	−0.681	1.514	0.202	0.653	0.506	0.026–9.844
CRP mg/l	−0.004	0.015	0.073	0.788	0.996	0.968–1.025

**Abbreviations:** *HOD* hospital day, *WBC* white blood cells, *IL-* interleukin-, *IFN-γ* Interferon-γ, *TNF-α* Tumor Necrosis Factor-α, *CD4* Cluster of Differentiation 4 receptors, *CD8* Cluster of Differentiation 8 receptors, *Th* helper T cell, *Ts* suppressor T cell, *NK* natural killer cell, *Neu* neutrophil, *PCT* procalcitonin, *CRP* C-reactive protein

### Potential implications of clinical mNGS test

#### Potential inappropriate antibiotic usage for patients with virus isolates

There were 4 viruses identified by mNGS from 23 patients in this study, the majority of the identified viruses were herpes simplex virus (*n* = 15), followed by Epstein-Barr virus/ herpes simplex virus (*n =* 5), Epstein-Barr virus (*n =* 1), Hepatitis A virus (*n =* 1) and torque teno virus (*n =* 1). Nearly 50% of patients were diagnosed with a hospital-acquired infection (12/23) and 17 of 23 patients were given broad-spectrum antibiotics based on symptoms, imaging. 10 of 23 patients were suspected of inappropriate antibiotic usage, which means after broad-spectrum antibiotic treatment, patients’ symptoms did not improve or even worsened and after identifying the real pathogen through mNGS and adjusting the antibiotic use based on that, patients’ condition improved. 7 of 23 were considered immunocompromised hosts characterized by deficiency of the immune system or immune response caused by infectious factors, mycotoxins, drugs and nutritional deficiencies. (Table 5).

**Table 5:** Clinical Characteristics of Patients with Virus Isolates (*n* = 23)

Type of Virus	HAI		Immunosuppressed Patients		Broad-spectrum Antibioticsa		Suspected Inappropriate Antibiotic Usage		Treatment Responsive	
	Yes	No	Yes	No	Yes	No	Yes	No	Yes	No
HSV (*n =* 15)	8	7	7	8	10	5	5	10	8	7
HAV(*n =* 1)	1	0	0	1	1	0	1	0	1	0
HSV/EBV (*n =* 5)	3	2	0	5	4	1	2	3	3	2
TTV(*n =* 1)	0	1	0	1	1	0	1	0	0	1
EBV (*n =* 1)	0	1	0	1	1	0	1	0	0	1
Total (*N =* 23)	12	11	7	16	17	6	10	13	12	11

**Abbreviations**: *EBV* Epstein-Barr virus, *HAI* hospital-acquired infection, *HSV* herpes simplex virus, *HAV* herpes simplex virus, *TTV* torqueteno virus

#### The influence of positive by mNGS on the hospital days and survival of patients

**Fig. 6:**
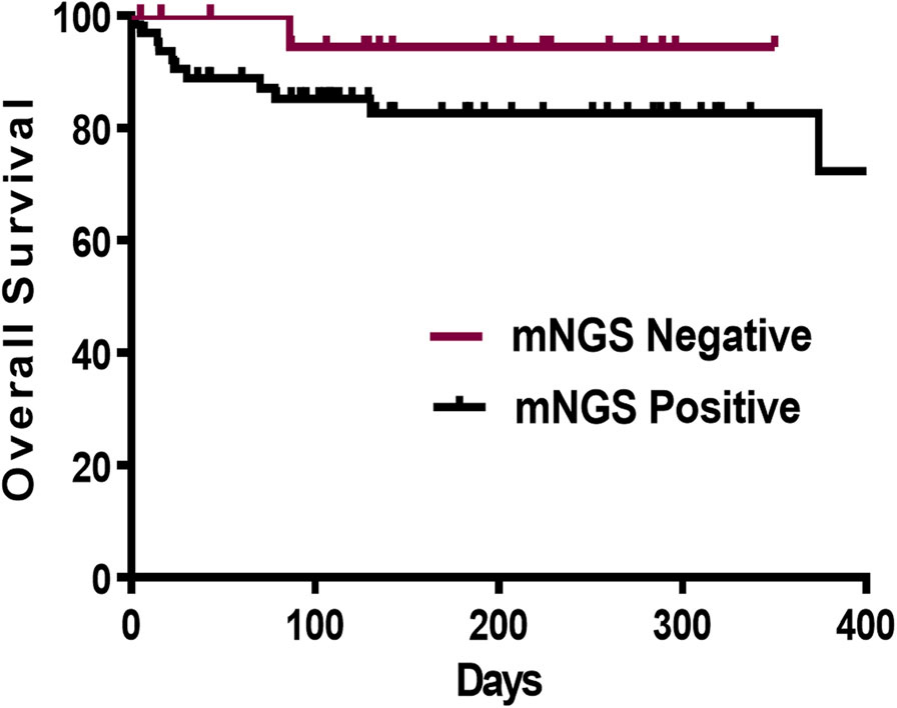
The survival curves of positive and negative group of mNGS in ID. The survival curves suggested that the overall survival rate declined faster in the positive group, however, there was no statistically differences between the two groups

As Table 6 showed, there were 67 samples in positive group with 57 males and 26 in negative group with 20 males. There was no significant difference in mean age between the two groups (59.70 yrs. vs 60.50 yrs., *P* = 0.84). Positive group had a longer hospital day (HOD, 176.63 days vs 150.96 days, *P* = 0.047) and a higher 28-day mortality (9.0% vs 0%, *P* = 0.049) than those of negative group, but there were no statistical differences in 14-day mortality (4.5% vs 0%, *P* = 0.278) and 90-day mortality (13.4% vs 3.9%, *P* = 0.180) between groups. The average survival time of two groups were 176.64 days and 150.96 days, respectively, but *P* value for t test between groups was 0.425, no statistical differences. The survival curves of the two groups were shown in Fig. 6. At the meantime, we analyzed the relationship between pathogens read number and HOD, 14-day-mortality, 28-day-mortality and 90-day-mortality, which showed that the higher pathogens read number, the higher 90-day-mortality and the longer HOD (Table 7).

**Table 6:** The basic demographic and clinical characteristics of initial and outcome patient variables

	Positive	Negative	P
Sex			
Female	10	6	0.355
Male	57	20	
Age	59.70 + 2.16	60.50 + 3.06	0.84
HOD	176.63 + 17.70	150.96 + 103.14	0.047
14 days of death	4.5%	0	0.278
28 days of death	9.0%	0	0.049
90 days of death	13.4%	3.9%	0.180
Read Number	5295.62 + 2507.26	16.67+ 4.79	0.039
Survival time	176.64 + 17.70	150.96+ 21.05	0.425

**Abbreviation:** *HOD* hospital day

**Table 7:** The analysis between the pathogens read number and HOD,14, 28 and 90-day-mortality

Read Number							
	0	1–9	10–99	100–999	1000-	F	P
No	20	15	20	24	14		
HOD	14.84 + 8.58	13.07 + 5.18	15.80 + 9.12	20.70 + 16.5	27.92 + 24.06	2.685	**0.037**
14-mortality	0	0	0	0.04	0.14	1.898	0.118
28-mortality	0	0.13	0.05	0.083	0.29	2.253	0.07
90-mortality	0.05	0.2	0.05	0.083	0.36	2.598	**0.042**
Survival Time	169.74 + 102.68	138.40 + 100.27	158.70 + 125.83	185.45 + 124.82	194.71 + 216.79	0.424	0.791

**Abbreviation:** *HOD* hospital day

## Discussion

The traditional clinical model for diagnosing infectious diseases is for doctors to make a differential diagnosis and then conduct a series of tests to try to identify the pathogen [9–12]. Traditional diagnostic techniques of microbiology laboratory ranges from smear microscopy, microorganisms’ culture, antigen antibody detection and PCR mainly. Whereas most traditional methods were often time-consuming and has a lower positive rate than mNGS [2–4]. Although molecular diagnostic assays are a quick way to diagnose the most common infections, almost all conventional microbial trials in use today only target a limited number of pathogens at a time or require successful culture of microorganisms from clinical samples [13]. While mNGS analyze the entire microbiome in patients’ samples [8] so it has been used to discover novel viral pathogens and diagnose viral infections in people widely [14–16]. Therefore, we explored the application and differences between traditional culture method and mNGS in clinical infectious diseases in adults. BALF, blood, sputum, tissue, CSF, pleural fluid, pus, bone marrow or nasal swab from 109 patients suspected of infection were collected and specimens were subjected to regular clinical microbiological assay and mNGS testing in a pairwise manner in our study. We then systematically compared the clinical features and test results of mNGS and traditional culture.

The results suggested that there were no significant differences in age, gender, length of stay and fatality rate between two groups and mNGS had an advantage in sensitivity rate compared with traditional culture method. A team of researchers also found that mNGS detected potential pathogenic bacteria, which had advantages in speed and sensitivity compared with culture and pathology [17], Miao’s team [5] showed that mNGS had a sensitivity of 50.7% for the diagnosis of infectious diseases, higher than traditional culture (50.7% vs 35.2%). In particular, the diagnosis of MTB, virus, anaerobic bacteria, nocardia and fungi has obvious advantages. The results were similar to our results, which showed that the sensitivity of mNGS was 67.4%, significantly higher than that of culture method (23.6%). High sensitivity of mNGS may because pathogen DNA has a long survival time in plasma, the use of antibiotics has a small impact on mNGS results, while traditional cultures are greatly affected by the use of antibiotics. Because of the small sample size, mNGS showed no statistical difference compared with culture method in pathogen types although there was a trend of superiority in Klebsiella, bacteria without MTB/NTM, EBV, CMV, NTM, Anaerobes, *Saccharomyces cerevisiae*, Proteus, Pneumocystis carinii, Abiotrophia, Nocardia, *Staphylococcus aureus*, Enterococcus and *Escherichia coli*. However, mNGS detection had a significantly higher sensitivity than the culture method in BALF (*P* = 0.002), tissue (*P* = 0.025), blood (*P* < 0.001) and sputum (*P* = 0.018) samples.

Based on the advantages shown by mNGS, we then investigated the influence of positive mNGS detection results on the severity and prognosis of patients with infection. By comparing the classification and counting of leukocyte, lymphocyte and cytokine concentrations in positive and negative groups, we found that IL-10 concentration in peripheral blood in the positive group was higher than that in the negative group and there were no statistically differences in other cytokine concentrations, leukocyte and lymphocyte. According to the results of correlative factors analysis for positive test of mNGS, patients’ age may promote the detection of pathogens. In the survival analysis, positive group had a higher 28-day mortality (9.0% vs 0%, *P* = 0.049) than that of negative group, but there were no statistical differences in average survival time. The pathogens read number by mNGS was positive related to the HOD and 90-day-mortality of patients with infectious diseases. All of that indicated older people were more likely to have positive results and positive results of mNGS detection may represent a worse outcome.

Fortunately, mNGS has moved from scientific application to clinical practice and is changing the way disease diagnosed and treated [18–20]. In addition to what we mentioned above, mNGS also has merits in many other aspects. Firstly, mNGS does not need prior clinical information to detect infectious pathogens, and the results can be reported quickly and accurately, greatly shortening the diagnosis time of infectious pathogens. Early and rapid reporting of the results by mNGS provides clinical clues to the next step in diagnosis and treatment, especially avoiding overuse of antibiotics for viral infections [21, 22]. Rapid results reported by mNGS also can promote timely adjustment of treatment in clinical practice. As our data showed, almost one-half of patients with virus infection were suspected of inappropriate antibiotic usage. Secondly, mNGS was used in some rare infectious pathogens. It detected *Naegleria fowleri* [23], brucellosis [24], cysticercosis, taenia bocinea [25], gondii [26] in CSF, Hepatic tuberculosis in blood [27] in previous reports. Thirdly, studies have shown that mNGS can be used not only for pathogen identification, but also for microbiome characterization, parallel analyses of human host responses, drug resistance gene and virulence factor detection. All of these led to the rapid development of mNGS in immunodeficiency difficult-to-diagnose cases and immunocompromised patients [13]. Thirdly, antibiotic usage had little influence on mNGS results due to the long survival time of pathogen DNA in plasma, but traditional cultures were affected by antibiotic use [21, 22]. Higher sensitivity of mNGS than culture in this study may because that mNGS is less affected by prior antibiotic usage. However, mNGS still has some limitations at present, such as human background, background bacteria contamination, no uniform standards for detailed experimental procedures [2, 28–31], inability to distinguish infection and colonization, standardization of bioinformatics analysis process, and problem of report interpretation. The results must be interpreted in the context of the clinical situation. It’s worth noting that background microbial contamination is a common problem faced by mNGS technology, which can be partially eliminated through negative quality control, but it requires clinical familiarity with common background bacteria and better interpretation results combined with clinical practice [24].

In this study, we systematically compared mNGS and traditional culture method in sensitivity, specificity, pathogen type and sample type. On this basis, we also compared and analyzed the differences between the positive and negative groups of mNGS which was few at present. Patients of positive group found to have a trend of worse prognosis suggested need more attention clinically. Small sample size was the biggest deficiency of our study, so that there were many results indicated a certain trend without reaching statistical significance unfortunately. Therefore, more patients need to be included in the study in the future. Not randomized controled was also the limitation of study. As a retrospective study, this study has some limitations like limited data and data accumulation not controlled by the researcher. Besides, limit generalizability caused by single-center study, lack of a gold standard comparator for diagnostics, lack of antibiotic usage detail and classification bias were also the limitations of this study.

## Conclusions

In summary, mNGS had a higher sensitivity than culture, especially in the types of BALF, blood and sputum samples, and there was a trend of higher sensitivity of Klebsiella, CMV and EBV detection. The worse trend of outcome in patients with positive mNGS results than negative group prompted more clinical attention to patients with positive mNGS results is required. Therefore, based on what we found above and other advantages of mNGS like quick results, less affected by prior antibiotic exposure and so on, we suggest that mNGS should be used more in early pathogen diagnosis in the future. Nonetheless, interpreting data of mNGS will be a challenge for doctors in guiding clinical treatment of infectious diseases.

## Data Availability

All data generated or analysed during this study are included in this published article. The data that support the findings of this study are available from the corresponding author upon reasonable request.

## Abbreviations

mNGS: Metagenomic next-generation sequencing
BALF: Bronchoalveolar lavage fluid
CSF: Cerebrospinal fluid
Dx: Diagnosis
ID: Infectious disease
NID: Noninfectious disease
DNA: Deoxyribonucleic acid
EDTA: Ethylene Diamine Tetraacetic Acid
EBV: Epstein-Barr virus
HAI: Hospital-acquired infection
HboV: Human bocavirus
HSV-1: Herpes simplex virus 1
TTV: Torqueteno virus
HAV: Hepatitis A virus
HOD: Hospital day
IL-2: Interleukin-2
IL-4: Interleukin-4
IL-6: Interleukin-6
IL-10: Interleukin-10
IL-8: Interleukin-10
CD4: Cluster of Differentiation 4 receptors
CD8: Cluster of Differentiation 8 receptors
WBC: White blood cell
Th: Helper T
NK: Natural killer cell
PCT: Procalcitonin
CRP: C-reactive protein
IFN-γ: Interferon-γ
IL-17a: Interleukin-17a
TNF-α: Tumor Necrosis Factor-α
PPV: Positive predictive value
NPV: Negative predictive value
CMV: Cytomegalovirus
MTB: *Mycobacterium tuberculosis*
ns: No signicant difference
NTM: Nontuberculous mycobacteria
G +: Gram Positive
G-: Gram Negative
